# Experiences Using Nonpharmacological Interventions for Chronic Fatigue: A Focus Group Study of Long‐Term Survivors of Young Adult Cancers With Fatigue

**DOI:** 10.1002/cnr2.2139

**Published:** 2024-09-05

**Authors:** Trine Stub, Marleen Mathisen, Lene Thorsen, Cecilie E. Kiserud, Hanne C. Lie

**Affiliations:** ^1^ NAFKAM‐Norway's National Research Center in Complementary and Alternative Medicine, Faculty of Health Science, Department of Community Medicine, UiT The Arctic University of Norway Tromsø Norway; ^2^ Department of Behavioural Sciences in Medicine Institute of Basic Medical Sciences in Medicine, Faculty of Medicine, University of Oslo Oslo Norway; ^3^ Department of Oncology, Division of Cancer Medicine Oslo University Hospital Oslo Norway

**Keywords:** chronic fatigue nonpharmacological interventions (NPI), facilitators and barriers, treatment programs, young adult cancer survivors (YACSs)

## Abstract

**Background:**

Cancer‐related fatigue is a common and distressing late effect of cancer that can persist for decades after treatment completion. Although negatively affecting survivors' quality of life, few, if any, efficacious interventions for persistent, or chronic, fatigue exist.

**Aims:**

To inform future interventions, we explored how long‐term, young adult cancer survivors (YACSs) with chronic fatigue live with, and manage their fatigue over time, including their experiences with nonpharmacological interventions (NPIs) for chronic fatigue.

**Methods and Results:**

We conducted a qualitative focus group study with 15 YACSs (13 women) with chronic fatigue, on average 7.3 years post‐diagnosis. The YACS were identified and recruited through a nationwide health survey of cancer survivors (the NOR‐CAYACS study). Systematic content analysis was used to identify recurrent themes. Analysis revealed five themes: (1) manifestation of fatigue, detailing chronic fatigue experiences; (2) impact on daily life, highlighting the necessity to balance rest and activity, affecting relationships; (3) NPIs, where walks in nature were notably beneficial; (4) barriers to fatigue management, including energy deficits, treatment‐related bodily changes, and self‐care prioritization challenges; (5) facilitators to fatigue management, emphasizing the need for regular breaks, self‐care practices, and the importance of fatigue management education.

**Conclusion:**

This study offers novel insights into the lived experiences of YACSs with chronic fatigue, a subject scarcely examined in prior research. Our findings highlight the significant impact of chronic fatigue and the individualized strategies YACSs use to cope. The research emphasizes the need for personalized interventions to support chronic fatigue management, marking a critical step forward in addressing this often‐overlooked issue in survivorship care. Future research should focus on tailored approaches to improve YACSs' quality of life.

**Abbreviations**
BMI: body mass index
CBT: cognitive behavior therapy
NPI: nonpharmacological interventions
YA: young adults
YACSs: YA cancer survivors

## 1 Introduction

Young adult cancer survivors (YACS), diagnosed between the ages of 18 and 39, represent a growing population due to great improvements in diagnostics and treatments and thus increased survival rates [1]. YACSs are at high risk of cancer‐related late effects [2], such as cardiopulmonary problems, hormonal disturbances, reduced fertility, second cancers, neurological sequelae, anxiety, and fatigue [3, 4].

Fatigue is one of the most common late effects, understood as a distressing, persistent, subjective sense of physical, emotional, and/or cognitive tiredness or exhaustion related to cancer diagnosis and treatment that is not proportional to recent activity and interferes with daily functioning [5, 6]. It can persist for years after treatment completion and greatly affects physical, psychological, and social function and health‐related quality of life [7, 8]. For YACS, who are at a life stage marked by educational attainment, establishing a family, and gaining employment and financial independence, fatigue can be especially challenging [9]. Despite this, research on the management of fatigue among YACS is scarce [10].

Chronic fatigue is defined as high levels of fatigue persisting for 6 months or more [11] was reported by 20%–29% YACS in a population‐based study among survivors of breast cancer, colorectal cancer, and nonHodgkin lymphoma at an average of 15 years after diagnosis [12], and among YACS of testicular cancer [13] and lymphoma [14].

Treatment options for chronic fatigue are limited, but nonpharmacological interventions (NPIs) appear to be more effective than pharmacological treatment options [15]. NPIs such as aerobic and/or strength training and/or psychological interventions (e.g., cognitive behavior therapy [CBT] [10, 16], patient education, and stress management [e.g., acupuncture, yoga, and mindfulness] [17]) have shown small to moderate effects on fatigue during and shortly after cancer treatment [15, 18].

A meta‐analysis by Hilfiker et al. found the effects of CBT combined with aerobic and/or resistance training, and yoga on fatigue in patients during and after cancer treatment [19]. In a review by Haussmann et al. yoga, psychosocial‐ and mindfulness‐based interventions were found beneficial on the level of fatigue, but psychosocial interventions had a larger effect than yoga and mindfulness [17]. Another study [10], found equal effects of exercise, psychoeducation, and mindfulness for managing fatigue.

So far, few studies have fatigue as the primary endpoint. Thus, most interventions are not specifically designed to improve levels of fatigue. Furthermore, studies evaluating the effects of NPIs among long‐term survivors with persisting fatigue at inclusion are lacking [20]. Additionally, there is a shortage of research, on how long‐term YACSs with chronic fatigue live with and manage their fatigue over time.

Qualitative research indicates that YACS often experience a liminal state, feeling neither fully ill nor completely healthy, which impedes their daily activities [21]. Chronic fatigue significantly restricts their participation in physical and social activities. Moreover, knowledge of the specific support needs of YACS beyond medical treatment and how they can connect with peers with similar experiences is currently limited [22, 23]. There is also a lack of evidence‐based knowledge about efficacious rehabilitation programs for managing chronic fatigue in cancer care in general. The specific needs of YACS and effective interventions for chronic fatigue among long‐term YACSs are poorly explored [24, 25]. Therefore, a qualitative inquiry into these issues can provide valuable insights when developing interventions to reduce fatigue and enhance the quality of life for the growing population of YACS with chronic fatigue.

We conducted a focus group study to explore how long‐term YACSs with chronic fatigue have managed their fatigue over time. Especially, we were interested in their experiences with the use of NPIs and their perceived barriers and facilitators to participate in fatigue‐related treatment programs.

## 2 Materials and Methods

### 2.1 Study Area and Setting

According to the Nordic health model, all citizens in Norway have access to universal health care, which is free of charge or highly subsidized [26]. YACSs is followed up by medical specialists, normally at their treating hospital through a standardized care program following national guidelines for the first 2 years after treatment completion. Beyond this, the general practitioner (GP) is the survivors' first port of call for health concerns.

### 2.2 Design

Four focus group interviews were conducted at the Department of Behavioural Medicine at the University of Oslo. Focus group design is particularly suited to studying lived experiences concerning specific topics of which there are spars previous knowledge [27, 28]. In this study, each participant received a group and an identification number to ensure anonymity.

### 2.3 The Interview Guide and Focus Group Interviews

The interview guide was developed based on a review of the literature and the research team's clinical experience. The interviews were semi‐structured and open‐ended, allowing the participants to give nuanced answers and follow‐up questions to be asked. The interview guide is included as Data S1.

### 2.4 Sample and Recruitment

Participants for the current study were identified and recruited from the population‐based health survey conducted in 2015/2016 among Norwegian Childhood, Adolescent, and YACSs (the Nor‐CAYACS study) (REC 2015/232) [29]. Participants were identified by the Cancer Registry of Norway and included all >5‐year survivors diagnosed between 1985 and 2009, with any childhood cancer (CCS, 0–18 years old, excluding central nervous system tumors), or breast cancer (stages I–III), colorectal cancer, leukemias, nonHodgkin lymphoma, or malignant melanoma at age19–39 years.

The Nor‐CAYACS questionnaire included Chalder's Fatigue Questionnaire (FQ), which measures mental and physical fatigue and allows for a score of chronic fatigue if severe symptoms have persisted for 6 months or longer [29].

For the current focus group study, YACSs reporting severe fatigue were identified. YACSs with more than one cancer diagnosis, distant metastases, ongoing treatment, and residency within more than a 1‐h drive of Oslo were excluded. Among 231 eligible YACSs with severe fatigue, 44 survivors were randomly extracted (using Microsoft Excel) and invited. Seventeen agreed to participate, two did not show up for the interview, and 15 completed the interviews. See Table 1 for participant characteristics.

**TABLE 1:** Characteristics of the participants.

	*N* = 15
Gender	
Women	13
Men	2
Mean age at interview (SD)	45.6 (5.7)
Education	
Upper secondary education (≤13 years)	6
Higher education (>13 years)	8
Missing	1
Marital status	
Living alone	2
With spouse no children	2
With spouse and children	7
Missing	4
Work	
Working 100%	6
Sickness leave	1
Work assessment allowance	2
Work part‐time	3
Disability pension	2
Missing	1
Age at diagnosis	
Mean (SD)	33.7 (5.4)
Diagnoses	
Breast cancer	9
Colorectal cancer	1
Lymphoma	4
Leukemia	1
Years since diagnosis	
Mean (SD)	7.3 (1.8)
Fatigue at Survey (mean (SD))	
Mental fatigue*	7.8 (1.6)
Physical fatigue**	13.3 (2.9)
Total fatigue***	21.1 (3.8)
Fatigue since diagnosis (yes)	14

*Note:* FQ includes 11 items, seven concerning physical fatigue and four mental fatigue. The total fatigue score*** ranged from 0 to 33, the physical fatigue score** from 0 to 21 and mental fatigue score* from 0 to 12. Severe fatigue 6 months or longer was defined as chronic fatigue [12].

### 2.5 Data Collection

Group interviews consisted of 2–6 YACSs. The interviews were audio‐recorded and transcribed *ad verbatim* [27]. The interviews were facilitated by two researchers (Hanne C. Lie and Lene Thorsen) experienced with chronic fatigue and survivorship care research. Hanne C. Lie, a psychologist experienced in conducting focus groups, facilitated the interviews. Lene Thorsen, an experienced sports physiologist was the observer. They did not know the participants prior to the interviews. The interviews lasted between 60 and 75 min and field notes were taken during the interviews.

### 2.6 Data Analysis

Two authors (Trine Stub and Marleen Mathisen) read the transcripts several times and created codes based on information from each group interview with input from the research team. Any disagreements were discussed until a consensus was reached. The analysis of the data was conducted according to conventional content analysis [30], allowing codes and themes to be identified in the data. Thus inductive coding was obtained [31]. A coding tree with 19 codes was developed, later merged into seven relevant themes due to identical information. Finally, five main themes were identified: *Manifestation of fatigue; impact on daily life*; *NPIs*; *barriers to fatigue management*; and *facilitators of fatigue management*. NVivo 1.61 [32] was used for data management and analysis.

## 3 Results

### 3.1 Characteristics of the Participants

In brief, the majority were women who had higher education but were not in full‐time work. Their mean age was 45.6 years. Breast cancer and nonHodgkin lymphoma were the most frequent cancer diagnoses.

### 3.2 Theme 1: Manifestations of Fatigue

All participants elaborated on how fatigue impacted their everyday lives, including physical and mental functioning at a personal and social level. Experiencing fatigue that persists years beyond treatment resulted in a distressing incongruence between their own and others' expectations of what they should be able to achieve versus what they were able to once they were “cured of cancer.” Some, but not all, of the participants, were aware that fatigue could be a late effect of cancer treatment, yet several found it hard to distinguish fatigue from what is “normal” tiredness and not. One participant said: *Don't know if it's the treatment or that I've got older* (*S1,3*). Despite this, they all had descriptions of how they experienced fatigue as described below.

### 3.3 Physical Symptoms

The participants described fatigue in terms of lack of energy; as having the flu with a gross and heavy body; with muscle aches and a feeling of lactic acid in their muscles; or like wearing a heavy blanket. As one explained:

I feel like I'm on my way down the drain when I have a shower. I'm so exhausted. My body is so tired that I'm wondering how on earth I will make it to work (S4,1).

Many participants confirmed that they were exhausted no matter how long they had slept, waking up with low charged battery:

I feel that I need more sleep than normal. I stay in bed for a long time. Sleep poorly maybe, but I'm always exhausted when I wake up (S1,1).

### 3.4 Mental Symptoms

Most participants were experiencing various forms of cognitive problems, such as poor memory and concentration. Moreover, they described that many chores take more time now than before, and that it is harder to make decisions, multi‐task, organize thoughts, and find the words. As explained: *I can talk to people about everyday things or I'm in a store to do shopping, and suddenly my mind goes blank* (*S3,4*).

Some described hypersensitivity to sounds and light, especially in busy surroundings. For some, it was exhausting to talk and relate to other people. Others described feeling worried, and restless with internal stress, as one described:

I feel there's no peace in my body. I feel that my body is a bit stressed. I'm very happy with my life and appreciate lots of things, but I feel more internal stress and I have many thoughts (S4,1).

### 3.5 Theme 2: Impact on Daily Life

The participants expressed that being constantly tired was extremely draining. To cope with daily life, they had to plan for breaks to regain energy. This felt frustrating because they expected life to return to the way it was before cancer. Many participants found it difficult to find the balance between rest and activity and to identify activities that gave or drained them of energy. One explained: *I should have worked less; I should have quit some of the voluntary work to do activities that would give me more energy* (S1.1).

As time passed and the fatigue persisted, they had difficulties coping with the situation and it affected their sense of self or self‐worth. One elaborated:

During the first couple of years, it was OK to blame it on the cancer treatment. The situation now is: Can I blame it on the treatment, or have I just become a lazy, useless person? Is that what has happened? (S2,4)

Others found it difficult to accept that they have fatigue.

The participants described that their energy levels varied from day to day and throughout the days, generating uncertainty regarding what they could plan and do. One participant illustrated fatigue through a metaphor:

I have a total of twelve teaspoons of energy when I wake up in the morning. One teaspoon equals one activity. You get up, shower, get dressed, and have breakfast. Then you have already used a third of the day's energy. Then you must allocate your energy to the obligations. You may have children, you may have a spouse, you may have a mother, you may have friends, and you may have things to do. Some days when I wake up, I might have three teaspoons. On other days when I wake up, I don't think there is more than half of one. Some days I might have twenty. I don't know this when I get up (S3,4).

This uncertainty and the general lack of energy resulted for many in feeling of loneliness and problems coping with unforeseen everyday events. One said:

I'm quite social, but I notice that I'm either completely on or completely off, and I don't have much energy. I increasingly enjoy sitting on the couch, but it's on and off (S2,2).

Some described the need for strict routines to best manage their everyday lives:

I need a routine for everything. So, my family occasionally suffers when I ask: Who put this here? It's supposed to be there! Everything needs to be in place for me to be able to exist. That's how it works for me, and it's the same at work, and I find this the most exhausting (S3,1).

They revealed how such “special needs,” and feelings of being lazy and ashamed of not being able to function as they and others expected, affected their inter‐personal relations often feeling like a burden to their families. One of the participants explained that she barely managed to get up on bad days and that she did not manage to empty the dishwasher. She remembered, “I just sat on the couch waiting for my partner to get home and fix things” (S4,2).

### 3.6 Theme 3: Nonpharmacological Interventions

The participants had two main aims concerning their health: to improve daily functioning and to function at the same level as before the cancer. They developed several strategies to achieve these aims over the years, which are summarized in Table 2 and described in the text below.

**TABLE 2:** Nonpharmacological interventions and self‐management strategies adopted by the participants.

Visits to a healthcare provider	Self‐help techniques	Physical/leisure activities	Self‐management strategies
Conventional healthcare personnel Doctors (*n* = 15)^a^ Nurses (*n* = 15) Physiotherapists (*n* = 2) Psychologists (*n* = 2) Complementary and Alternative Medicine (CAM) providers Coach (*n* = 1) Kinesiologist (*n* = 1) Acupuncturists (*n* = 1) Official healthcare system Rehabilitation stays (*n* = 1)	Breathing techniques (*n* = 1) Mindfulness (*n* = 1) Meditation (*n* = 1)	Physical activities Being outdoors in nature (*n* = 5) Hiking, jogging, walking (*n* = 1) Training/exercises at the gym/Taekwondo (*n* = 4) Paddling (*n* = 1) Swimming (*n* = 1) Hobbies Gardening (*n* = 1) Singing in a choir (*n* = 1)	Self‐care practices Value of being a human (*n* = 1) Positive attitude (*n* = 1) Connect the head and body (*n* = 1) Balance your energy throughout the day(*n* = 1) Know your limits (*n* = 1) Prioritize rehabilitation stays (*n* = 1) Daily routines for improving everyday life Do not rush (*n* = 1) Take breaks and rest (*n* = 1) Eat well and establish routines (*n* = 1) Use sticky notes to remember tasks (*n* = 1) Get help from family and friends (*n* = 3) Work reduced hours (*n* = 1) Be physically active (Start slowly and improve step by step) (*n* = 1)

^a^ Number of participants who reported these strategies.

### 3.7 Visits to Healthcare Providers

The participants needed someone to talk to about their health issues and learn more about how to best cope with fatigue. They wanted providers who were friendly, knowledgeable, patient, and respectful. They saw GPs and talked to nurses, CAM providers, and other health personnel (e.g., physiotherapists and psychologists) for help and to get insight into their situation. One participant (S2,3) saw a psychologist and CAM providers to “clear her head and talk.” During the consultations, it was confirmed that fatigue was not unusual after cancer, and the therapists encouraged her to have confidence in her strategies to deal with fatigue herself: *It's better to see a therapist than get a lot of advice from friends and colleagues, that might not work and leave me frustrated* (S2,3).

The participants had various experiences seeing their GP, but most were pleased with their GP who provided good help and support:

My doctor was fairly inexperienced when I got ill, so she worked very hard to check and follow up. She did a great job (S4,3).

Other participants hardly ever saw their GPs because they were unsure whether the doctor had sufficient competence in the late effects (including fatigue) of cancer treatment.

*Often when you ask your doctor, they refer you to your oncologist who treated you at the hospital. They* (*the GPs*) *regard the oncologists as the experts, so you don't get much help* (S1,2).

### 3.8 Self‐Help Techniques

Because of fatigue, many struggled with internal stress and restlessness. To reduce these complaints, and indirectly improve their fatigue, they used various mindfulness and meditation approaches. As explained by one:

At least I get started very slowly (with mindfulness), and you learn how to relax, and it also helps you sleep better (S3,1).

Other participants have also had positive experiences with mindfulness and breathing techniques to reduce stress and anxiety attacks, as explained:

I have done some mindfulness and have not taken any medication (S2,3).

Now the heart can beat slower, I don't have to think about anything, so I can just breathe, breathe (S4,1).

One participant meditated each morning and evening for 20 min for the last 3 years. The technique has helped him cope with anxiety about recurrence and negative thoughts about his fatigue. He told: *The more distance, the more I relax, and I've spent some time learning to accept that this is life. However, it just takes a lot of conscious effort* (S5,1).

### 3.9 The Benefits of Leisure Activities

Leisure activities were regarded as important for managing fatigue and increasing a general sense of well‐being. The most popular leisure activity among all participants was walking. They went walking when they needed some peace and fresh air. Some found walking better than exercising at the gym and others enjoyed walking the dog. In the forest, no one demanded anything of them, as one participant experienced:

I like the forest very much, but I think it's also because it's so quiet there. It's my place in a way. Some of my friends often want to walk with me, and sometimes that's nice, but very often I think it's better to walk alone. It's not because I don't want to be with them or be social, it's just that I can't handle it as I need to boost my energy, and then I can't listen to all sorts of everyday problems. I need to protect myself (S2,2).

Other leisure activities that helped “re‐charge their batteries” were gardening and singing in choir: *I've started singing in a choir, which has been an odd experience for me. When I go there, I can be exhausted and worn out. When I get back home, I have lots of energy. So, my experience has been that laying on the couch doesn't necessarily boost your energy. Energy can also be found elsewhere* (S2,3).

#### 3.9.1 Self‐Care Practices

After many years with fatigue, the participants have found ways of coping with it. To avoid making fatigue worse, it was important to balance physical activities with rest and avoid draining activities. Through experience, they had learned ways to distribute their energy throughout the day. As a participant experienced: *When the children are at school, I go to the gym. Then, I must rest before the children get home, so I have the energy to help them with homework and so on. My days have been like this for almost ten years now* (S1,4).

The participants claimed that it was important to keep the body going but to start slowly to avoid getting worn out, but that it could be challenging to maintain activity: *When your head says one thing and the body something else, you end up living with two different parts, and you cannot relate to the body* (S3,4).

Many had painful experiences with getting worn out after too much activity and after such incident one participant was offered rehabilitation. She explained:

I learned (at the rehabilitation stay) not to rush, but rather be physically active, eat healthy foods, and have good routines. So, I've had my routines ever since, and when I don't stick to my routines, I get worse. I understand that it's very important to keep my body going (S2,4).

In rehabilitation, cancer survivors also learned how to change old habits to manage their fatigue. One explained: *In the course, we learned that we had to connect the head and body. It's almost impossible to change habits on your own, so we got much help* (*from healthcare professionals at the rehabilitation center*) (S3,4).

Further advantages of rehabilitation stay on their general wellbeing were to learn to accept and value the “new version” of oneself: One of the most important things I've learned is the value of being a person. It's not about what I do, but what I am, and we don't learn that. From early childhood, we have learned that we're assessed on what we do. And suddenly you can't do that any longer, you are just you. This perspective has been most helpful for me (S4,3).

Several participants mentioned writing notes and using other techniques to help them remember, structure, and organize their life.

I have written “train” on my hand today. That's no problem, but I go by bus every day from work. Today I will go by train. Then I write a reminder on my hand to avoid having to spend energy trying to remember. But I don't know if it has anything to do with the treatment. No, that's just a peg to hang it on, but I experience that my memory is quite different. I use yellow stickers and have a system so people don't notice (S2,2).

### 3.10 Theme 4: Barriers to Fatigue Management

A common barrier to engaging in physical activity was changes to their bodies and how it responds to exercise after cancer. One of the participants did a lot of exercise before he got cancer and started exercising immediately after treatment. He said: *When I'd had my last chemotherapy, I ran straight out in the forest. That didn't work too well* (S4,2).

He repeatedly tried running but struggled to find the optimal exercise intensity. Therefore, he gave up. Similarly, another participant who was a fitness instructor was accustomed to a high level of activity before being diagnosed. Nevertheless, after the cancer treatment, she had to try a lot more to achieve the same training effect as before. She explained:

So, I need to work a bit harder than my friends. They benefit more easily from training. But I've been wondering if it has to do with the cancer or the fact that I've got older. So, I can't quite figure out what's what. But it's much harder to keep in shape now, and it requires much more effort (S1,3).

Another barrier to physical activity was that even just getting out the door required precious energy: *My problem is getting out the door, so I must exercise at home or go for a walk* (S3, 4).

The weather also played an important role, with winter posing particular challenges. One participant said:

It works quite well to exercise in the summer, but in the winter, I don't have the energy. I don't stand a chance and feel guilty (S3,4).

For some, it was also difficult to prioritize their own needs in an otherwise busy life, as this participant explained:

I think exercising has been good and worked well for a long period. When you must prioritize your energy, it's easy to put yourself last. At least that's been the case for me (S2,2).

Lack of information, knowledge or access to advice regarding how to exercise when their energy was low was another barrier. As one participant expressed:

Some suggestions from a personal trainer on how to do strength training, as strength is also good for the body, isn't it? And advice on what to look out for, what's good and what's not (S4,1).

Further, as another explained: *I thought I was informed, but not very thoroughly. They* (*physicians at the treating hospital*) *merely referred to some websites. I don't remember exactly how it was, but I had to figure it out myself* (S1,3).

The participants wanted a consultation about fatigue and training sometime after treatment completion when they had experienced “how their bodies worked”:

The best for me would have been if they had asked me how I was doing when I went to the hospital for control and when I mentioned that I was fatigued they could have given me some suggestions and options. Not only an option, but correct and valid information about what this is (S3,4).

### 3.11 Theme 5: Facilitator of Fatigue Management

The participants had a great desire to go back to the way it was before cancer diagnosis and treatment. However, it was easier to cope with fatigue and facilitate participation in activities when they accepted that they were not like before and needed rest. After having relaxed on the couch enjoying a book, one of the participants thought: *I should do this more often* (S4,1). Another facilitator was to think positively about their new situation or avoid compare their “new lives” to how life used to be as that could make them sad, depressed and inactive.

The participants had different types of motivation for participating in NPIs for fatigue. One of the participants, exercised because it made her feel better and as such a part of her self‐care. She said: *I don't exercise to get physically stronger, but I feel better up here* (*mentally*). *If I skip exercise for an entire week, I get in a bad mood. It gets a bit easier, and I get more energy if I can squeeze exercise into my schedule, regardless of how motivated I feel when I go there. It still helps and maybe I appreciate that part more now than before I got cancer. I feel I need it more now* (S1,1). However, she did not push herself as hard as she used to.

### 3.12 The Internet

Facebook was a source of information about various offers and information to cancer survivors. The participants were members of groups where they could post their questions. This was perceived as useful. *When you sit there in the middle of the night wondering about something, you can post a question. There's always someone awake there to answer your questions. Many of us have problems in sleeping. It was of great help to me* (*S3, 2*).

Others have received links about cancer, fatigue, and exercise from friends and colleagues. One of the participants searched on the internet for exercises one could implement by self *The only information I found was that you should exercise no matter how you feel. But I'm not very fond of exercising, but I like to go for a walk* (*S5,1*).

## 4 Discussion

Participants in this study experienced chronic fatigue, as they described a lack of energy, body heaviness, and muscle pain, in addition to hypersensitivity, and cognitive difficulties. They faced challenges in balancing rest and activity, negatively impacting relationships.

This study contributes with new and in‐depth insight into, how long‐term YACS with chronic fatigue live with and manage their fatigue over time. The participants believed that the anti‐cancer treatment had negatively changed their body and body image, leading to deep self‐awareness and reflection processes.

Based on experience and through trial and error, they have discovered activities and strategies that alleviate their fatigue. These strategies include practicing self‐care that is, balancing the energy throughout the day, prioritizing one's own needs, positive attitude about the new situation, the value of being a human, and knowing their (physical and mental) limits. To function well in everyday life, they have learned to take breaks and not rush, establish routines, and be physically active. Walking in nature was the most used strategy by the participants, providing mental, and physical rejuvenation.

Barriers to participating in activities included physical changes from cancer treatment, the fatigue itself and lack of energy, difficulty prioritizing self‐care, and weather conditions. Participants emphasized the need for information and education on fatigue management.

### 4.1 Other Studies

The present study participants experienced both physical and mental fatigue. They described fatigue as a lack of energy, accompanied by a heavy feeling in the body and muscle pain. They also had heightened sensitivity to sounds and light, as well as difficulties with memory and concentration. These findings are consistent with a previous study that found high levels of emotional and cognitive fatigue among YACS of breast and gynecological cancer [9].

Chronic fatigue negatively affected the participants' relationships, as they lacked the energy to socialize, and had difficulty coping with unexpected events. The study's findings align with previous research that showed YACSs with and without chronic fatigue to be more socially connected but experience greater loneliness compared to noncancer controls [33]. Additionally, a Norwegian study [21] revealed that YACSs often felt stuck between being neither ill nor healthy and struggled to participate in daily activities. They expressed a desire to be understood and actively involved in society [21]. However, qualitative studies exploring social support among YACSs found that friends provided general support and helped them feel like normal teenagers despite their cancer diagnosis. These friends played a crucial role in providing social and emotional support during their cancer journey [34, 35].

To the best of our knowledge, this study is the first to investigate chronic fatigue among YACS, and thus, there are no directly comparable studies available among physical/leisure time activities, walking in nature emerged as the most potent strategy, providing a tranquil environment that revitalizes the participants mentally and physically.

Among participants in our study, physical changes after cancer treatment, weather, lack of energy, and difficulty prioritizing their own needs hindered their ability to be physically active. These findings are in line with a study in 2015 that reported that fatigue itself was a significant barrier to physical and social activities [36]. These findings are consistent with the research conducted by Bøhn et al. [12], who found that nonadherence to lifestyle guidelines was associated with having chronic fatigue [12]. Moreover, a review by Caru et al. [16], highlighted that integrating physical activity into the lives of YACS posed challenges due to busy schedules and travel requirements, family or work demands, lack of time, health issues, and pain. Psychological barriers, fatigue itself, lack of knowledge/misconceptions about physical activities, and physical limitations due to condition and/or treatment were identified in another study by Adamovich et al. [37].

Participants in our study emphasized the importance of information and education in managing fatigue. They expressed a lack of such resources. However, previous research on psychoeducation and educational interventions has shown no statistically significant effect on chronic fatigue compared to other coping strategies [38].

Further, self‐care was viewed as crucial to managing fatigue and participating in activities, along with maintaining a positive attitude toward life. The participants in this study also emphasized the importance of having a flexible attitude toward making necessary changes. The participants acknowledged that they were not the same individuals as before and recognized the need to prioritize daily breaks and time‐outs to better cope with chronic fatigue and other daily tasks. However, finding the right balance between rest and activity was challenging for them. This finding is in line with Gibson et al. [39], who found that parents and healthcare providers (59%) recommended rest and relaxation to handle chronic fatigue.

### 4.2 Practical Implications

This study has identified useful insights for developing tailored symptom management interventions targeting chronic fatigue in YACS. Incorporating NPIs (such as CBT) alongside physical activities, including aerobic and resistance training, as well as yoga and mindfulness in recommended intervention formats and strategies would be in line with the participant's experiences and preferences. Physical activity interventions involving walking in nature, either alone or in groups, would likely cater to individual preferences for stress reduction and/or alleviating loneliness [23]. Additionally, participants lacked information on managing and living with late effects; thus, incorporating patient education and self‐management seems essential in such interventions. Moreover, making interventions multi‐dimensional, tailored to individual needs, and flexible, would allow participants to take breaks or switch activities as needed.

### 4.3 Limitations

Data were collected from YACS recruited through a national survey with a 39% response rate, suggesting potential selection bias. However, the risk of nonresponse bias in the study was statistically examined using existing data on every individual in the entire population provided by the Cancer Registry of Norway and found to be minimal [29]. Moreover, regarding the recruitment in the current study, using a purposive sampling strategy, rather than randomly selecting participants from a list of 231 eligible individuals, could have enhanced participant diversity in terms of gender, ethnicity, age, and diagnosis, potentially offering a broader range of perspectives [31]. As such, the under‐representation of such is an important limitation to the transferability of our results.

### 4.4 Strengths

Focus group interviews provide a comprehensive understanding of the participants' experiences with chronic fatigue and their efforts to adapt to life with this late effect. The participants exhibited similarities in their statements, which reinforced the validity of the findings [31]. Data saturation was achieved after four group interviews, indicating that additional interviews did not yield new information.

Further research involving larger and more diverse samples of YACS with chronic fatigue would be beneficial to enhance the generalizability and robustness of the results.

## 5 Conclusion

This study sheds light on the experiences of YACSs with chronic fatigue, illustrating its various symptoms and the challenges it poses in daily life. Several nonpharmacological modalities were used to handle chronic fatigue, which were selected and combined individually. Further interventions should include modalities customized to each person's needs and preferences. In addition, the participants emphasized the need for information and education on fatigue management.

## Author Contributions

**Trine Stub:** writing – original draft, methodology, software, writing – review and editing, formal analysis, data curation, validation. **Marleen Mathisen:** methodology, writing – review and editing, software, formal analysis, data curation. **Lene Thorsen:** conceptualization, investigation, methodology, writing – review and editing, project administration, supervision, validation, resources. **Cecilie E. Kiserud:** conceptualization, writing – review and editing, methodology, project administration, supervision, validation, resources. **Hanne C. Lie:** conceptualization, investigation, methodology, writing – review and editing, supervision, resources.

## Ethics Statement

The southeast regional committee for medical and health research ethics (REC 2015/232) and the data protection officer at Oslo University Hospital have approved the study. All participants signed informed consent before inclusion. Consent for publication was obtained from the participants. This study was reported according to the consolidated criteria for reporting qualitative studies (COREQ): a 32‐item checklist [40].

## Conflicts of Interest

The authors declare no conflicts of interest.

## Supporting information


**Data S1.** Supporting Information.

## Data Availability

The data are not publicly available due to Norwegian privacy regulations. Applicants for any data must prepare to conform to Norwegian privacy regulations. Researchers who want to request the data can contact the last author.
