# Supplementary material for: Experiences Using Nonpharmacological Interventions for Chronic Fatigue: A Focus Group Study of Long‐Term Survivors of Young Adult Cancers With Fatigue
Source: Cancer Rep (Hoboken). 2024 Sep 5;7(9):e2139. doi: 10.1002/cnr2.2139 (PMC11375328; doi:10.1002/cnr2.2139)
Supplement: Supplementary file 1 — Data S1. Supporting Information. [file CNR2-7-e2139-s001.docx]

Information about the project

- Background
  - research project
  - aim to improve health services to cancer survivors with persistent fatigue
  - enhance knowledge of applicable treatments
  - The findings from this interview will be published in articles and at conferences.

Formalities

- Duty of confidentiality and anonymity
- A conversation in which there are no right/wrong answers
- The role and background of the investigator/interviewer
- Get informed consent of recording the group interview
- Inform how the recording will be used and stored

Ask if anything is unclear and if the participants have any questions

The text in cursive is merely help for the investigators/interviews. It is not necessary to ask all the questions.

1. **Please tell me about yourself and your present situation.**
2. **What is your experience with fatigue?**

*When did it start?*

*Change over time?*

*What kind of information did you get about fatigue? Who gave you this information?*

1. **How does fatigue affect you as a person?**

*Physically (fitness/strength)*

*Mentally/Cognitively (memory, planning, concentration)*

1. **How does fatigue affect your daily activities?**

*Indoor and outdoor activities (housework, work, social, exercise)*

1. **How was life before you got ill?**

*Indoor and outdoor activities.*

1. **Have you tried to reduce the fatigue/tiredness?**

*On your own/Organized offers*

*GP/Other health personnel*

*Stay physically active/Exercise offers/Rehabilitation stay*

*Mindfulness/Other psychological measures to reduce stress/mental health services/Diet/Lifestyle*

| **If yes** | **If no** |
| --- | --- |
| 1. What was their motivation for participating? | What would have been their motivation for trying? |
| 1. Did you accomplish your goal/Did you complete the program? |  |
| 1. What made it hard/easy to accomplish your goal? (personal/social) | What would have made it hard/easier to try? (Personal, social) |
| 1. What was the benefit? |  |

We know that adapted physical and/or mental exercise might reduce fatigue. However, many people cannot use such services for various reasons. Therefore, we would like to know if you would welcome such services. If yes, how could such services be adapted to your life.

1. **What do you think of such services?**
2. **Based on your experience, what would such services include and how should they be structured?**

*Start with an open question, then make a separate list of suggestions for the content and structure of such services (yes/no questions).*

1. **What do you think would increase the chances for people with long-term fatigue to try and manage to go through such a treatment offer (personal/social)?**
2. **Finally, what kind of advice would you give others in the same situation?**

**Summary of main themes that have been addressed.**

1. Did we understand you correctly?
2. Anything else you would like to add?
